# Association between weather variables and fascial space infections of the head and neck: a retrospective chart review

**DOI:** 10.1186/s12903-025-06621-y

**Published:** 2025-08-12

**Authors:** Teresa E Fowler, Ryan F Bloomquist, Jeffrey N James

**Affiliations:** 1 Department of Ophthalmology, Augusta University, Augusta, 30912 USA; 2 University of South Carolina School of Medicine, Columbia, SC 29209 USA; 3 Department of Oral and Maxillofacial Surgery, Louisiana State University, New Orleans, 70119 USA; 4 Residency Program in Oral and Maxillofacial Surgery, LSUHSC School of Dentistry, 1100 Florida Avenue, New Orleans, LA 70119 USA

## Abstract

**Background:**

The incidence of fascial space infections of the head and neck has long been suggested to correlate with weather patterns, though objective evidence is inconsistent, and a causative effect is difficult to prove. One issue in the existing literature is that correlations between space infections and weather patterns have been assessed in only a few climates, so data from additional regions is needed. The purpose of this study is to understand whether head and neck infection rates correlate with weather patterns in a humid subtropical climate, adding to the current literature derived largely from temperate environments.

**Methods:**

In this retrospective observational study, we investigate potential associations between weather variables and fascial space infections of the head and neck treated by oral and maxillofacial surgery (OMFS) at Augusta University Medical Center, an urban tertiary care center in the Southeastern United States, over a 7-year period. The study sample included all patients presenting between October 2012 and September 2019 for head and neck infections identified by ICD10 code. Those with pre-existing oral or maxillofacial disease, including recent infection, were excluded. Daily weather reports from this period were obtained from the National Oceanic and Atmospheric Administration online database. The primary outcome variable, “infection rate” was stratified according to the day, month, year, and season of diagnosis. Spearman correlation coefficients were calculated for infection rate with each weather variable in each time grouping, with needle plots constructed to visualize trends. Statistical significance was determined with p-value < 0.05.

**Results:**

199 patients were included. Only when infection rates were stratified by year did Spearman correlations reach statistical significance. Correlations that reached statistical significance included daily departure from normal average temperature, cooling degree days, daily maximum dry bulb temperature, average dry bulb temperature, average wet bulb temperature, and daily minimum dry bulb temperature. Using the null hypothesis that there was no correlation between weather variables and infection rates, all other correlation coefficients failed to reach statistical significance.

**Conclusions:**

The results of this study do not support an association between weather and head and neck fascial space infections in the southeastern United States.

**Supplementary Information:**

The online version contains supplementary material available at 10.1186/s12903-025-06621-y.

## Introduction

Fascial space infections of the head and neck are a common reason for oral and maxillofacial surgery (OMFS) consult. Primary infection of the teeth, periodontal tissues, or surrounding bone can infiltrate the potential spaces within the head and neck bound by connective tissue layers. Once access to these planes is gained, infection can spread rapidly within the affected compartment, risking injury to important nerve and vascular structures enclosed within. Severe infections can lead to life-threatening airway compromise, sepsis, intravascular emboli or thrombi, or infection of nearby tissues such as the heart and lungs. Common microorganism culprits include those present on the skin and in oral flora including aerobic *Streptococcus*,* Staphylococcus*, and *Klebsiella* species, as well as anaerobes such as *Prevotella*,* Proprionibacterium*,* Bacteroides*, and *Fusobacterium* [1–3]. Antibiotics and surgical drainage are the mainstays of treatment.

As climate change has gained public attention over the last half century, medical providers have sought to understand the impact of weather on human health. In the specific case of head and neck infections, conflicting findings have been reported. One large study of 19,218 patients in Austria demonstrated a significant rise in surgically-managed dental abscesses in periods of low barometric pressure, while no correlation was identified in regard to temperature [4]. Ristow et al. report a weak correlation between low barometric pressure and odontogenic abscess in 1,211 patients in Germany, and no correlations with lunar phase, humidity, precipitation, or sunshine hours [5]. Additional evidence demonstrates associations between odontogenic abscesses and atmospheric pressure [6] or temperature [7], however separate studies did not support these associations [8, 9]. These results varied, although all of these studies were performed in Western Europe, a region with temperate mid-latitude climate. We wanted to know if a correlation between head and neck infections and weather could be identified in a humid, subtropical climate in the southeastern United States.

Though similar studies have been conducted previously in other parts of the world, there is a lack conclusive evidence from which to confirm or deny correlations between odontogenic infection and specific weather variables. The purpose of the present study was to determine whether the incidence of head and neck fascial space infections treated at a single tertiary care center in Georgia correlates with local weather patterns.

## Materials and methods

### Study design/sample

To address the research purpose, investigators designed a retrospective observational study with data collection performed by chart review. Due to the retrospective and deidentified nature of this study with minimal risk to study subjects, this study was exempt from Augusta University Institutional Review Board oversight. The study population was composed of all patients diagnosed by the OMFS service with head and neck fascial space infections at Augusta University Medical Center between October 1, 2012 and September 30, 2019. To be included in the study sample, patients must have been diagnosed with a head and neck fascial space infection involving the superficial, deep, or visceral fascial spaces. Patients were excluded as study subjects if they had pre-existing or newly diagnosed concomitant oral or maxillofacial disease, such as malignancy, osteoradionecrosis, or infection treated with antibiotics within the prior month. We identified patients based on ICD10 codes, including all patients diagnosed with an infectious process of the head and neck based on oral and maxillofacial surgeon criteria using a combination of clinical and/or imaging data.

### Data collection

The number of head and neck infections diagnosed for each time period was obtained using ICD-10 codes. Weather data was obtained from the National Oceanic and Atmospheric Administration online database at https://www.ncei.noaa.gov/cdo-web/ [10]. Statistical analysis was performed using SPSS software (version 25, IBM Corp., 2017). Data for continuous variables was summarized using mean, median, and standard deviation calculations. The associations between continuous variables were analyzed using spearman correlation coefficients.

### Variables

The primary outcome variable, “infection rate”, was calculated according to each time grouping by adding up the total number of head and neck infections during that time unit (month, year, etc.) and dividing by the total number of days in the time period. For example, when infection rate was stratified by month, the total number of diagnosed infections for each month was tabulated and divided by the number of days in that specific month.

The following weather measures were used as secondary predictor variables: average dew point temperature, average dry bulb temperature, average relative humidity, average sea level pressure, average station pressure, average wet bulb temperature, average wind speed, cooling degree days, departure from normal average temperature, heating degree days, maximum dry bulb temperature, minimum dry bulb temperature, peak wind direction, peak wind speed, precipitation, snow depth, snowfall, sustained wind direction, and sustained wind speed. Snow depth and snowfall were later removed from the analysis because measurable amounts of snow fell in Augusta on only 4 days out of the 2,555 days between October 1, 2012 and September 30, 2019.

### Data analysis

Infection and weather data was stratified into several time groupings, including day of the week (Sunday - Saturday), month of the year (January - December), year (2012–2019), season (spring, summer, fall, winter), and “month-year” (October 2012– September 2019). Needle plots were constructed for infection rate and each of the weather variables by time period in order to identify trends.

Spearman’s correlation coefficients were calculated to evaluate the relationship between infection rate and each weather variable in each time period grouping. For example, correlation coefficient was calculated between each weather variable summarized according to month with the infection rate also summarized according to month for direct comparison. This analysis was repeated for each variable and unit of time studied. A significance level of 0.05 was used for all two-tailed tests unless otherwise specified.

## Results

**Fig. 1:**
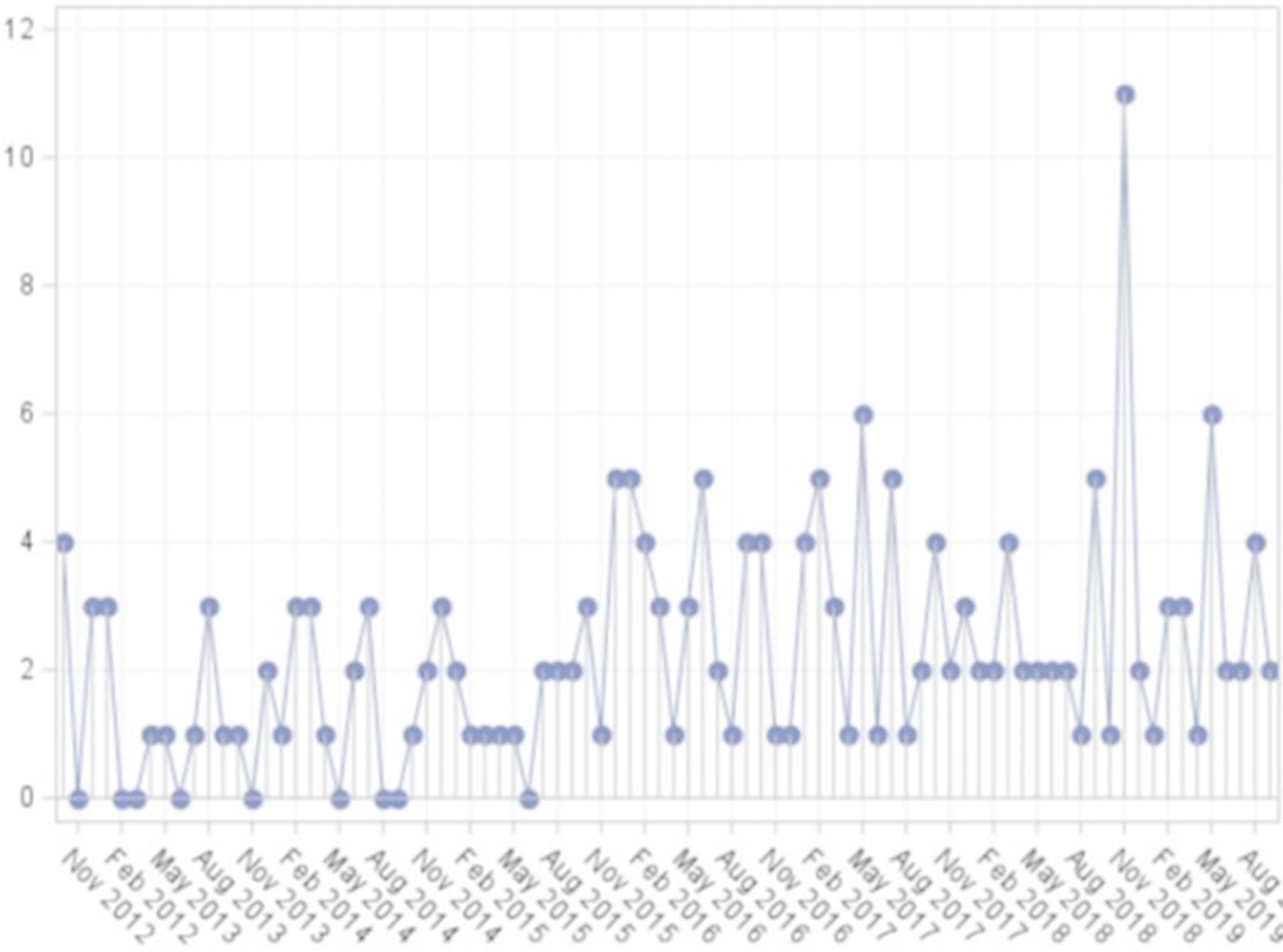
Needle plot of the monthly number of head and neck infections starting in October 2012 and continuing through September 2019. A data point is present for each of the 84 included months, through for clarity the x-axis is only labeled every 3rd month. Of note, there was a statistically significant spike in November 2018 relative to the 6 months before and the 6 months after though no unifying factor could be identified

A total of 199 patients met inclusion criteria for analysis. The daily head and neck infection rate calculated using all 2,555 days of data was 0.07. We first summarized weather variables by month-year (*n* = 84 months), calculating the mean, standard deviation, minimum, and maximum displayed for each variable (Supplemental Table 1). Calculations were next made based on year (*n* = 8 years) (Supplemental Table 2). Needle plots were created in order to observe temporal trends in individual variables. In Fig. 1, the number of infections diagnosed is plotted for each month from October 2012 to September 2019. Of note, a statistically significant spike was observed in November 2018 relative to the 6 months prior and 6 months after, with 11 infections diagnosed (*p* < 0.05 for each monthly comparison).

**Fig. 2:**
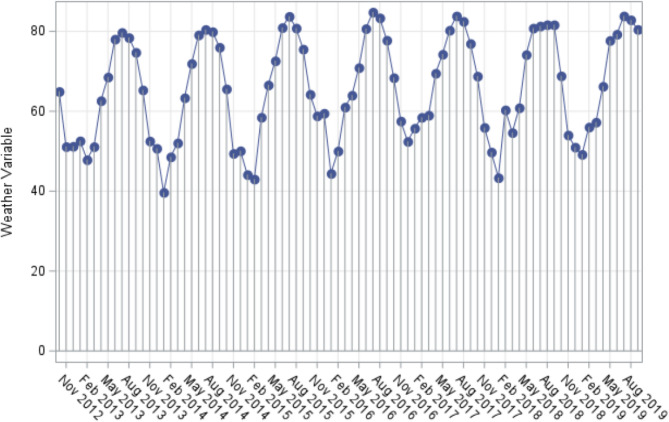
Needle plot for Average Dry Bulb Temperature. The expected seasonal cyclical variation is evident from the plot. A data point is present for each of the 84 included months from October 2012 through September 2019, through for clarity the x-axis is only labeled every 3rd month

**Fig. 3:**
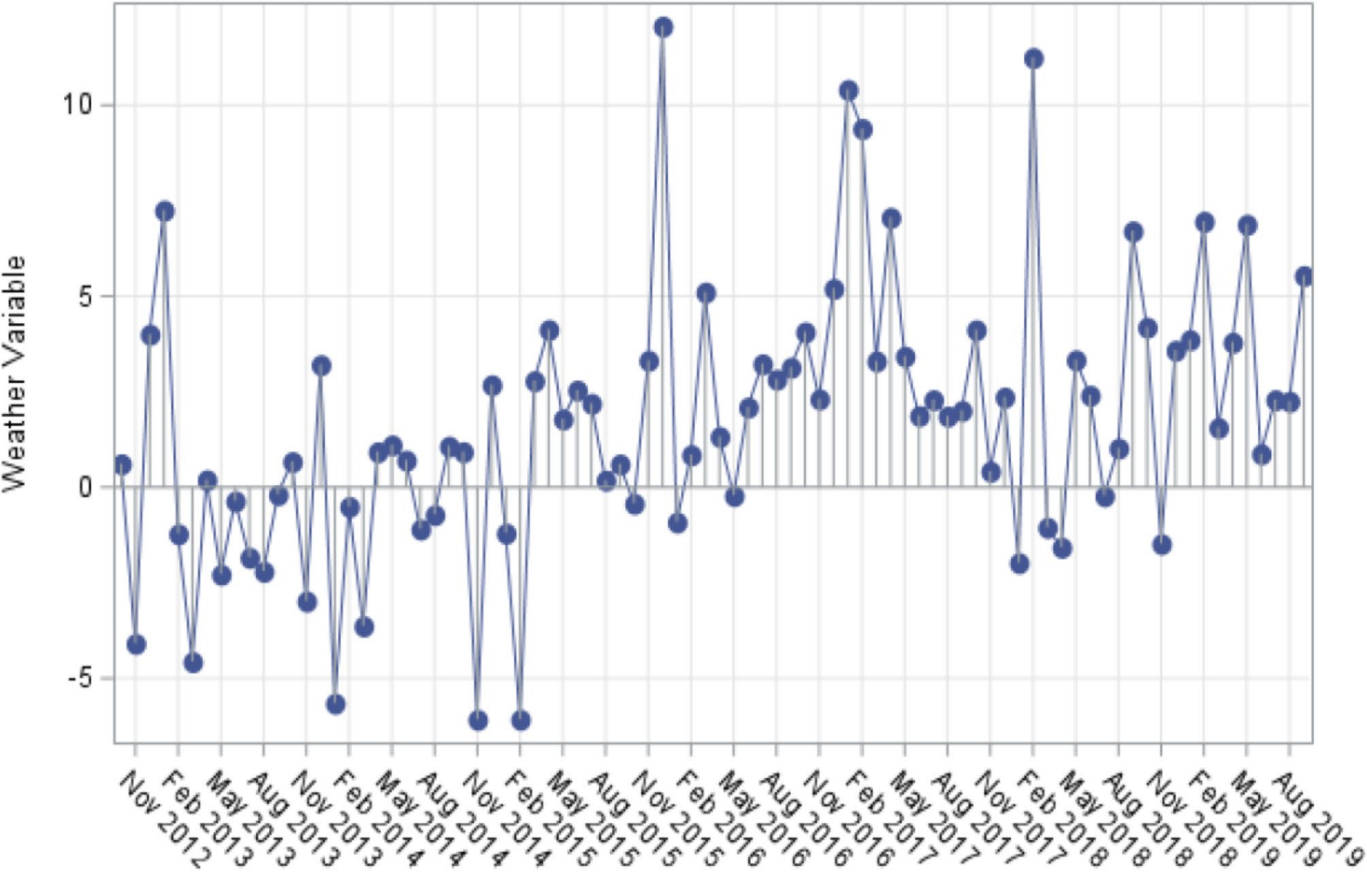
Needle plot for Departure from Normal Average Temperature. This needle plot demonstrates seemingly random peaks and nadirs with no identifiable pattern. A data point is present for each of the 84 included months, through for clarity the x-axis is only labeled every 3rd month

Needle plots created for each of the weather variables by month-year demonstrated expected cyclical variation. For example, Fig. 2 demonstrates the needle plot for average dry bulb temperature. As expected, temperature peaked during the summer months of June, July, and August, with troughs during the winter months of December, January, February for each year. Variables with less predictability, such as departure from normal average temperature (Fig. 3), did not show a strong trend, with seemingly random peaks and troughs throughout the time period plotted. We evaluated and compared each plot for trends. For example, November 2018 demonstrates a negative deviation for departure from normal average temperature (*p* < 0.05 compared to the month prior and the month following), the same month in which there was a large spike in the number of head and neck infections diagnosed.

Spearman correlation coefficients were calculated between infection rates and weather variables, and the only statistically significant correlations identified were when stratified by year. All statistically significant correlations are demonstrated in Table 1 with corresponding p-values. Variables with statistically significant correlations, listed in descending order of strength of relationship, included daily departure from normal average temperature (r_s_ = 0.88, *p* = 0.004), cooling degree days (r_s_ = 0.29, *p* = 0.021), daily maximum dry bulb temperature (r_s_ = 0.74, *p* = 0.037), average dry bulb temperature (r_s_ = 0.71, *p* = 0.047), average wet bulb temperature (r_s_ = 0.71, *p* = 0.047), and daily minimum dry bulb temperature (r_s_ = 0.71, *p* = 0.047).

**Table 1:** Statistically significant spearman correlations between weather variables and infection rate, yearly summaries

Weather Variable	*n*	*r*_s_	*p*-value
Daily Departure from Normal Average Temperature	8	0.88	0.004
Cooling Degree Days	8	0.79	0.021
Daily Maximum Dry Bulb Temperature	8	0.74	0.037
Average Dry Bulb Temperature	8	0.71	0.047
Average Wet Bulb Temperature	8	0.71	0.047
Daily Minimum Dry Bulb Temperature	8	0.71	0.047

r_s_: Spearman correlation

**Fig. 4:**
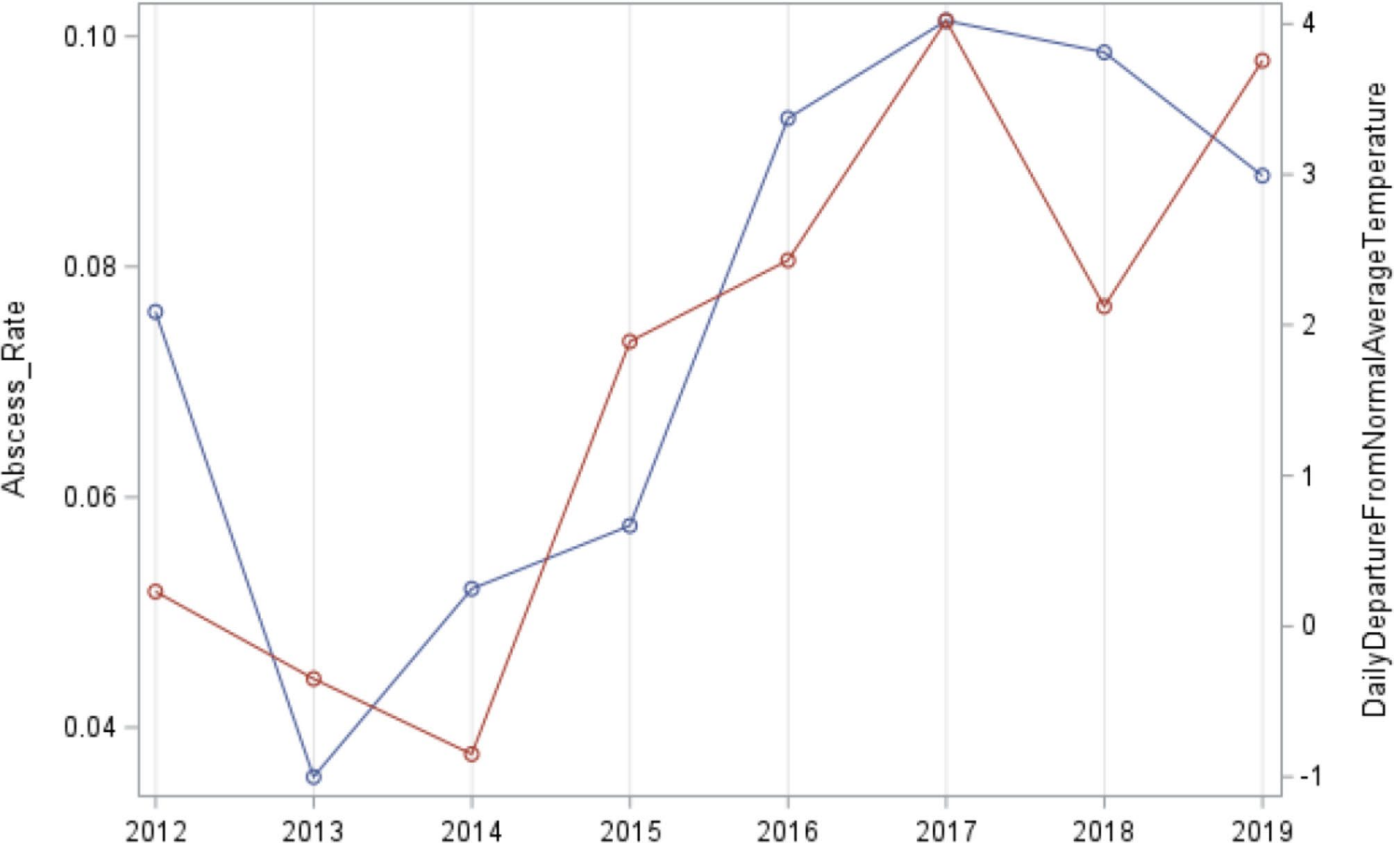
Plots of the yearly series for the infection rate and the yearly series for Daily Departure from Normal Average Temperature. The x axis depicts the year, from 2012 to 2019. The red line denotes the infection rate for each year, with the y axis on left ranging from 0.04 (minimum) to 0.10 (maximum). The blue line depicts the daily departure from normal average temperature, with y axis on the right ranging from − 0.8 (minimum) to 4.0 (maximum). The trends between the two series over time are very similar, resulting in the strong Spearman correlation coefficient (r_s_ = 0.88, *p* = 0.004)

Figure 4 plots infection rate and average daily departure from normal average temperature by year on the same graph. The changes over time in the two series are similar, resulting in the strong Spearman correlation of 0.88. Comparison of the same two variables by any other time period stratification (day, month, month-year, season) did not yield any significant correlations.

Since there were only two months of weather data available for 2012 (November and December), Spearman correlations were repeated after omission of 2012 data. When examining only 2013–2019 (*n* = 7) as shown in Table 2, the correlation between infection rate and cooling degree days (r_s_ = 0.89, *p* = 0.007) was stronger than that of daily departure from normal average temperature (r_s_ = 0.86, *p* = 0.014). The remainder of correlations were similar to those calculated with 2012 data included, with slightly overall stronger correlations. An additional correlation was identified between infection rate from 2013 to 2019 and daily average dew point temperature (r_s_ = 0.79, *p* = 0.036).

**Table 2:** Statistically significant spearman correlations between weather variables and infection rate, yearly summaries after omitting 2012

Weather Variable	*n*	*r*_s_	*p*-value
Cooling Degree Days	7	0.89	0.007
Daily Departure from Normal Average Temperature	7	0.86	0.014
Daily Maximum Dry Bulb Temperature	7	0.82	0.023
Average Wet Bulb Temperature	7	0.82	0.023
Daily Minimum Dry Bulb Temperature	7	0.82	0.023
Daily Average Dew Point Temperature	7	0.79	0.036
Average Dry Bulb Temperature	7	0.79	0.036

r_s_: Spearman correlation

## Discussion

This study addresses a gap in knowledge regarding possible correlation between head and neck fascial space infections and weather variables in a subtropical climate in the southeastern United States. The risk of odontogenic head and neck infection is affected by individual oral flora [11], as well as anatomic elements such as the ligamentous and fascial connections between dentition, bone, and muscle of the face [12]. Given the complex interplay between factors, a relationship between weather and infections is difficult to pinpoint. Unfortunately, there are no large location-specific data sets available for tracking these correlations, and published data regarding odontogenic infections and weather variables is inconsistent.

While overall correlations identified in our study were weak, statistically significant associations emerged when infection rates were analyzed by year, particularly with temperature-related factors. Our findings align with previous studies that found no statistically significant effect of air temperature [4, 7, 9, 13] or atmospheric pressure [9] on head and neck infection frequency. However, they contrast with studies reporting associations between odontogenic infection and barometric pressure [4–6] or felt temperature [7]. These discrepancies between reports may stem from differences in study design, sample size, statistical analyses, or interpretation. For example, the studies by lead authors Keller [7], Seemann [4], Ristow [5], and Spalthoff [8] included only patients treated surgically, while Tarle included odontogenic abscesses requiring hospitalization [6]. In our present study, all patients diagnosed with head and neck infection during the target period were included.

We also note the possibility that these differences reflect a scientific phenomenon. It is possible that weather-infection associations are more pronounced in specific climates due to true variation in local human and microbial characteristics. For example, differences in oral microbiota have been shown between regions of the northwestern United States and Germany [14], and between groups of varying ethnicity [15, 16]. Additional factors may also contribute to the observed variations. Healthcare related factors such as patient transfer patterns, physician access, local health culture, and regional antibiotic usage could affect the patient cohort seeking treatment. Additionally, geographic climate variations may influence the microbial environment and susceptibility to infection. For example, warmer temperatures have been linked to increased risk of urinary tract infections [17, 18], cellulitis [19–21], tick borne diseases [22, 23], and increased virulence of several viral pathogens [24, 25].

The sample size, and therefore power, of this study is limited by its retrospective nature. While no statistically significant association was found in our analysis, a larger patient cohort may provide additional insight. Like other studies of its kind, we do not have the ability to account for individual patient factors, such as daily activities, exposures, or time spent outdoors versus indoors. It is possible that some of these patients traveled to areas with differing weather patterns when their infections were contracted but were later diagnosed and treated in Georgia, or were transferred from outside hospitals. Additionally, we did not link infection data with patient comorbidities, demographics, nor infectious organism, all of which would be of interest in this discussion to ascertain whether correlations result from host, climate, or microorganism properties. With increasing public focus on environmental contributions to human health and disease, we expect that more information will come to light regarding health metrics and weather variables.

## Conclusion

Overall, this study provides a new dataset for humid, subtropical areas to add to those published in more temperate climates. While yearly associations between temperature variables and infection rates were identified, they were not universally consistent when stratified by different time periods. This retrospective study does not identify a significant association between weather and head and neck fascial space infections in the southeastern United States, but further research is needed to better clarify these relationships.

## Electronic supplementary material

Below is the link to the electronic supplementary material.

Supplementary Material 1

## Data Availability

No datasets were generated or analysed during the current study.

## Abbreviations

OMFS: Oral and maxillofacial surgery
