# Supplementary material for: Association between weather variables and fascial space infections of the head and neck: a retrospective chart review
Source: BMC Oral Health. 2025 Aug 12;25:1322. doi: 10.1186/s12903-025-06621-y (PMC12344879; doi:10.1186/s12903-025-06621-y)
Supplement: Supplementary file 1 — Supplementary Material 1 [file 12903_2025_6621_MOESM1_ESM.docx]

**Supplemental tables**

**Supplemental table 1:** **Descriptive Statistics for Infection Rate and Weather Variables, by Month-Year**.

| Variable | n | Mean | Std. Dev. | Min | Max |
| --- | --- | --- | --- | --- | --- |
| Infection Rate | 84 | 0.07 | 0.06 | 0.00 | 0.37 |
| Daily Precipitation | 84 | 0.13 | 0.08 | 0.01 | 0.42 |
| Daily Average Dew Point Temperature | 82 | 53.5 | 13.8 | 24.0 | 72.7 |
| Daily Average Dry Bulb Temperature | 84 | 65.6 | 13.0 | 39.7 | 84.8 |
| Daily Average Relative Humidity | 82 | 71.0 | 6.1 | 54.8 | 83.8 |
| Daily Average Sea Level Pressure | 82 | 30.1 | 0.1 | 29.9 | 30.3 |
| Daily Average Station Pressure | 84 | 29.9 | 0.1 | 29.8 | 30.1 |
| Daily Average Wet Bulb Temperature | 82 | 58.5 | 12.2 | 33.4 | 75.5 |
| Daily Average Wind Speed | 84 | 5.1 | 1.0 | 3.3 | 7.7 |
| Daily Cooling Degree Days | 84 | 6.5 | 6.9 | 0.0 | 19.8 |
| Daily Departure From Normal Average  Temperature | 84 | 1.7 | 3.5 | -6.1 | 12.1 |
| Daily Heating Degree Days | 84 | 5.9 | 6.9 | 0.0 | 25.3 |
| Daily Maximum Dry Bulb Temperature | 84 | 77.8 | 12.5 | 52.7 | 97.5 |
| Daily Minimum Dry Bulb Temperature | 84 | 53.0 | 13.6 | 26.3 | 72.9 |
| Daily Peak Wind Direction | 84 | 193.1 | 30.4 | 111.3 | 260.3 |
| Daily Peak Wind Speed | 84 | 21.3 | 2.4 | 15.4 | 26.9 |
| Daily Sustained Wind Direction | 84 | 196.4 | 31.8 | 118.0 | 267.1 |
| Daily Sustained Wind Speed | 84 | 16.1 | 1.9 | 11.9 | 19.8 |

**Supplemental table 2: Descriptive Statistics for Infection Rate and Weather Variables, by Year**

| Variable | n | Mean | Std. Dev. | Min | Max |
| --- | --- | --- | --- | --- | --- |
| Infection Rate | 8 | 0.08 | 0.02 | 0.04 | 0.10 |
| Daily Precipitation | 8 | 0.12 | 0.02 | 0.08 | 0.15 |
| Daily Average Dew Point Temperature | 8 | 52.8 | 4.2 | 43.6 | 56.5 |
| Daily Average Dry Bulb Temperature | 8 | 64.9 | 4.3 | 55.8 | 70.5 |
| Daily Average Relative Humidity | 8 | 71.0 | 1.9 | 69.2 | 73.6 |
| Daily Average Sea Level Pressure | 8 | 30.1 | 0.0 | 30.0 | 30.1 |
| Daily Average Station Pressure | 8 | 29.9 | 0.0 | 29.9 | 29.9 |
| Daily Average Wet Bulb Temperature | 8 | 57.9 | 3.9 | 49.3 | 61.5 |
| Daily Average Wind Speed | 8 | 5.1 | 0.2 | 4.6 | 5.3 |
| Daily Cooling Degree Days | 8 | 6.1 | 2.5 | 0.9 | 9.4 |
| Daily Departure From Normal Average  Temperature | 8 | 1.7 | 1.8 | -0.8 | 4.0 |
| Daily Heating Degree Days | 8 | 6.2 | 1.9 | 4.0 | 10.1 |
| Daily Maximum Dry Bulb Temperature | 8 | 77.1 | 4.2 | 68.6 | 82.9 |
| Daily Minimum Dry Bulb Temperature | 8 | 52.2 | 4.5 | 42.5 | 57.5 |
| Daily Peak Wind Direction | 8 | 192.8 | 4.5 | 187.8 | 198.5 |
| Daily Peak Wind Speed | 8 | 21.1 | 0.8 | 19.2 | 21.8 |
| Daily Sustained Wind Direction | 8 | 196.1 | 5.5 | 190.5 | 207.0 |
| Daily Sustained Wind Speed | 8 | 16.0 | 0.6 | 14.5 | 16.4 |
